# Optimized Micro-Pattern Design and Fabrication of a Light Guide Plate Using Micro-Injection Molding

**DOI:** 10.3390/polym13234244

**Published:** 2021-12-03

**Authors:** Fang-Yu Fan, Hsin-Hua Chou, Wei-Chun Lin, Chiung-Fang Huang, Yi Lin, Yung-Kang Shen, Muhammad Ruslin

**Affiliations:** 1 School of Dental Technology, College of Oral Medicine, Taipei Medical University, Taipei 11031, Taiwan; fish884027@tmu.edu.tw (F.-Y.F.); weichun1253@tmu.edu.tw (W.-C.L.); chiung0102@tmu.edu.tw (C.-F.H.); 2 School of Oral Hygiene, College of Oral Medicine, Taipei Medical University, Taipei 11031, Taiwan; hhchou@tmu.edu.tw; 3 Division of Family and Operative Dentistry, Department of Dentistry, Taipei Medical University Hospital, Taipei 11031, Taiwan; 4 Department of Business Administration, Takming University of Science and Technology, Taipei 11451, Taiwan; linyi@takming.edu.tw; 5 Department of Oral and Maxillofacial Surgery, Faculty of Dentistry, Hasanuddin University, Makassar 90245, Indonesia; mruslin@unhas.ac.id

**Keywords:** illuminance, uniformity, processing parameter, light guide plate, micro-injection molding

## Abstract

This study examined the uniformity of illuminance field distributions of light guide plates (LGPs). First, the authors designed microstructural patterns on the surface of an LGP. Then, a mold of the LGP with the optimal microstructural design was fabricated by a photolithography method. Micro-injection molding (μIM) was used to manufacture the molded LGPs. μIM technology can simultaneously manufacture large-sized wedge-shaped LGPs and micro-scale microstructures. Finally, illuminance values of the field distributions of the LGPs with various microstructures were obtained through optical field measurements. This study compared the illuminance field distributions of LGPs with various designs and structures, which included LGPs without and those with microstructure on the primary design and the optimal design. The average illuminance of the LGP with microstructures and the optimal design was roughly 196.1 cd/m^2^. Its average illuminance was 1.3 times that of the LGP without microstructures. This study also discusses illuminance field distributions of LGPs with microstructures that were influenced by various μIM process parameters. The mold temperature was found to be the most important processing parameter affecting the illuminance field distribution of molded LGPs fabricated by μIM. The molded LGP with microstructures and the optimal design had better uniformity than that with microstructures and the primary design and that without microstructures. The uniformity of the LGP with microstructures and the optimal design was roughly 86.4%. Its uniformity was nearly 1.65 times that of the LGP without microstructures. The optimized design and fabrication of LGPs with microstructure exhibited good uniformity of illuminance field distributions.

## 1. Introduction

Light guide plates (LGPs) are a major component of liquid crystal displays (LCDs). An LGP uniformly transfers light from a cold cathode fluorescent lamp (CCFL) to the face of an LCD. The fidelity of the microstructures of the LGP and its optical properties are essential to ensure a good performance.

Many methods of manufacturing LGPs have been developed, including an ultraviolet (UV)-based imprinting process on large and thin LGPs with a dot-size [1], replication of LGPs with microstructures by injection molding (IM) [2,3,4,5,6], and a hot-embossing method [7,8,9,10]. Hot embossing, nano-imprinting, UV-embossing, compression molding, and IM are low-cost mass-production methods suited to replicate LGP’s microstructures. However, the E-beam, focused ion beam, and modified lithografie, galvanoformung, abformung (LIGA) techniques are expensive, complex, and not easily accessible to many scientists and industrialists.

There are many research papers emphasizing the brightness of LGPs with various processing parameters during molding. Lee and He [11] proposed that a new LGP with two different micro-structural V-cut surfaces provided higher brightness and uniformity. Kim et al. [12] investigated light intensity distributions of a 7-in (17.8 cm) LGP to increase the uniformity of the output illuminance using reducing bright and dark areas by a plurality of light emitting diodes (LEDs). Liu et al. [13] developed a gray relational analysis and fuzzy logic to achieve optimization of multi-response characteristics. Illumination was improved from 328.387 to 343.751 cd/m^2^, and the homogeneity increased from 63.21% to 72.65%. Li et al. [14] proposed a neural-network optical model for a backlight module (BLM) of an LCD (13 in or 33.9 cm) to expedite the design of light-scattering prism-pattern of its LGP. The luminance uniformity of the LCD BLM reached 93.1%. Pan and Fan [15] proposed a hybrid BLM with a hybrid light guide and a brightness enhancement film (BEF). Pan and Hu [16] designed an LGP with periodic, single-sized microstructures. Teng [17] proposed a method of “digital laser-blastering” (DLB) to fabricate a large-sized LED LGP with high luminance and efficiency. The average luminance of DLB LGPs increased 103~122% compared to a control group. Yang and Yang [18] presented hot-embossing for replicating continuous and discontinuous V-grooved microstructures on double-sided large-area substrates. Chung et al. [19] demonstrated an LGP by CO_2_ laser LIGA-like technology including laser-ablated microstructure of polymethyl methacrylate (PMMA) molds and a polydimethylsiloxane (PDMS) casting. The maximum luminance of 119 cd/m^2^ occurred at a taper angle of 40°. Lu et al. [20] analyzed the polarization-preserving property of two conventional edge-lit LGPs based on scattering dots and refractive microgrooves. Xu et al. [21] emphasized a BLM with an integrated micro-optical LGP. The uniformity of the luminance of the output light reached 93%. Hong et al. [22] investigated the effects of adding rapid heat-cycle injection-compression molding (RICM) to the optical anisotropy of a molded LGP. Wang et al. [23] investigated the effects of surface treatments, driving voltages and distances between the nozzle and substrate on various shapes and sizes of inkjet print droplets from polyacrylamide solution on PMMA substrates. The uniformity of the LGP was 83.85% with an average luminance of 1012.58 nit. Wang et al. [24] designed a high-directional backlight with special microstructures. Compared to a conventional BLM, the novel configuration achieved higher uniformity (of >90%). Kuo et al. [25] determined that the optimal processing parameters in a reciprocal comparisons approach were a cooling time of 30 s, a mold temperature of 85 °C, a melt temperature of 250 °C, an injection speed of 195 mm/s, an injection pressure of 240 MPa, a packing pressure of 110 MPa, packing switching of 5 mm, and a packing time of 3 s. Joo and Ko [26] studied the micro-prism pattern negatively inscribed into an LGP’s bottom surface, which was found to be the most effective design. Xu et al. [27] indicated that the luminance uniformity of a BLM depended on the microstructure distributed on the LGP’s bottom surface. The luminance uniformities of integrated BLMs exceeded 85%. Yoon et al. [28] designed local dimming technology for integration with LCDs in order to improve their contrast ratios. Meng et al. [29] developed a multi-layered waveguide LC smart window with full-color rendering. Chen et al. [30] indicated that the approach would have great potential and broad prospects for polarization-related LGP and mini-LED backlights. Quesada et al. [31] fabricated an all-glass, lenticular lens array and light guide substrates in a single masking and etching procedure. These structures’ aspect ratios and pitches effectively controlled the degree of light confinement (>80%) along the lenticular corridors. Min et al. [32] conducted a series of IM experiments to understand the distribution of yellowness in injection-molded LGPs and changes in optical properties under various IM conditions. Lee et al. [33] synthesized micro hollow plate-type silica, which was applied as an optical structure to develop a light-diffusion material that satisfied requirements of high transmissibility and luminance. Yu et al. [34] addressed the optical design of LED edge-lit LGPs for a front light unit panel with high illuminating contrast. Ye et al. [35] designed traditional backlights with new dot patterns, and IM, laser beam fabrication, or UV roll-to-plate imprinting applied dot patterns onto the LGP. The experiment achieved an efficiency of 85% and uniformity of 92.6%. Yoo et al. [36] presented a retinal-projection-based near-eye display with switchable multiple viewpoints by polarization-multiplexing. Feng et al. [37] evaluated the influence of heat radiation, heat conduction, and plastic deformation, a novel temperature model of the injection rolling zone during continuous-injection direct rolling. The average light transmittance was 88.32% and average reflectance was 8.7%. Wu et al. [38] designed and manufactured a composite LGP with a double-sided structure that significantly increased the central average brightness and uniformity of luminance. Nie et al. [39] demonstrated a novel liquid-level sensor based on a designed LGP. Wang et al. [40] developed the microstructures of various shapes that were engraved with a CO_2_ laser onto a glass LGP’s surface. Jiang et al. [41] developed the dimensions of an edge-lit LGP had a non-negligible impact on its output performance based on a pre-determined micro-dot array. Wang et al. [42] created an excellent LGP using variable scattering dots to replace simplex dots. Huang et al. [43] proposed grayscale direct-write lithography to process three-dimensional micro-nano structures, in order to fabricate two-dimensional distributed micro-prism arrays onto an LGP’s bottom surface. Liu et al. [44] compared femtosecond laser-etched microstructures and BLM optical performances of K9 glass and PMMA. The luminance uniformity of the K9 glass LGP was 90%, which was greater than that of the PMMA LGP. Wang et al. [45] improved the optical performance of an LGP using pyramid-shaped microstructures on the LGP’s bottom surface. The average luminance and luminance uniformity of LGP were 2352.8 cd/m^2^ and 92.4%, respectively. Quesada et al. [46] investigated all-glass, micro-groove arrays etched into a glass LGP surface. Luminance uniformities in excess of 80% for none-point and 75% for 455-point measurements were achieved with 1.2% extraction efficiency per groove.

In this work, the objective of the LGPs was to uniformly transfer light to the face of an LCD. The microstructures of the LGP and its optical properties were evaluated to ensure a good performance. We fabricated an LGP using a novel and effective procedure. We first designed the sizes of the microstructure on the LGP’s surface. The mold insert of the LGP with an optimal design of microstructure was manufactured by a photolithographic method. Finally, μIM was used to fabricate the LGP. An experimental study was conducted to characterize the effects of various μIM process parameters on the molded LGP. The optical properties of the molded LGP were measured and analyzed. In this way, the authors achieved an optimal illuminance field distribution of the molded LGPs.

## 2. Materials and Methods

We are the first to conduct a microstructural design of an LGP. When we obtain the best uniformity of an LGP with microstructures then this situation can be used as a mold design for manufacturing as in the second part of the study. The measurement method was based on the light emitted by a cold cathode fluorescent lamp (CCFL) through a self-designed fixture, and an added reflector to assemble a BLM; we applied an illuminance measurement method to measure the influence of diffusion point designs of various microstructures on the uniformity of LGP, and employed the Taguchi method to find the optimal combination of the arrangement of the microstructure distribution, and design the microstructural form of the LGP according to the best uniformity. The second part consists of the mold design and fabrication of the microstructure of the wedge-shaped LGP. The designed microstructure of the LGP used a photolithographic method to etch the microstructures of the LGP onto the metal mold. Finally, we used the metal mold to manufacture the molded LGP with microstructures by μIM. The optimal uniformity of illuminance field distributions of the LGP with microstructures was affected by various μIM process parameters.

**Figure 1:**
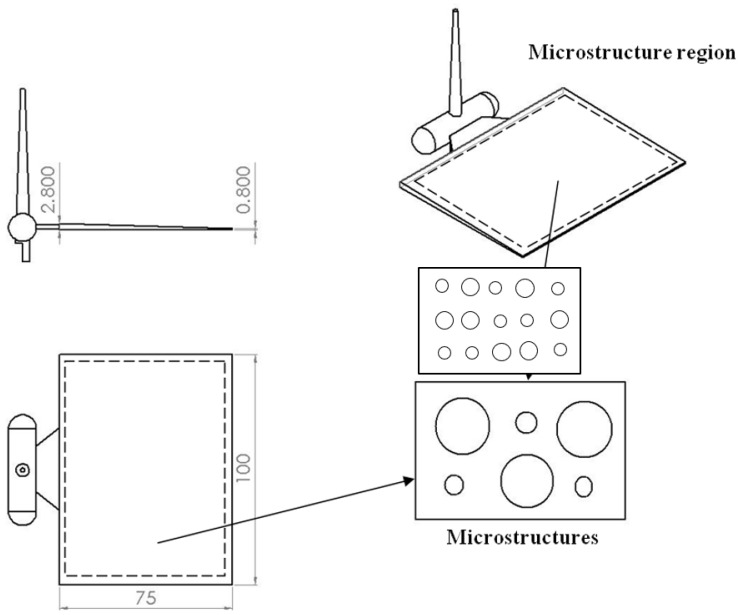
The dimensions of light guide plate. (unit: mm).

**Figure 2:**
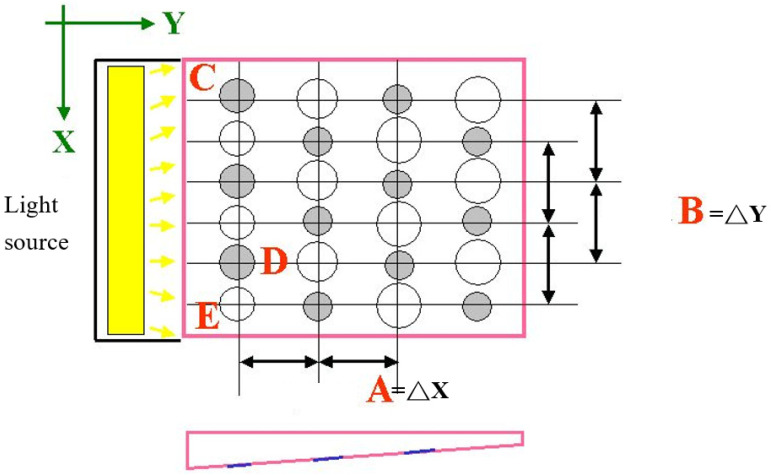
Stagger and insertion pattern of diffusion points of microstructure of light guide plate.

The dimensions of the LGP and its surface patterns (microstructures) are shown on Figure 1. We investigated the arrangement of a staggered insertion pattern of diffusion points at the microstructure of the LGP (Figure 2). The design parameters were the distance in the ΔX-direction (A), the distance in the ΔY-direction (B), the existence of the insertion point (C), the size of the circular shape of the diffusion point at the intersection point on the thick end (D), and the size of the circular shape of the diffusion point at the thick end (E). Table 1 lists the values of design parameters for the pattern of diffusion points on the LGP with microstructures. This study used five parameters and two levels to construct an L8 experiment (2^5^). The eight experiments of design parameters of microstructures of the LGP are described in Table 2. In Table 2, a ninth experiment for an LGP without microstructures was added to compare to the eight experiments of LGPs with microstructures. The design of the diffusion points was as follows. (1). The diffusion points were plotted using AutoCAD (computer-aided design) software (Autodesk, 2020). (2). We used a laser printer to print the plotted diffusion points onto a slide. (3). The uniformity of the light of the printed slide (with plotted diffusion points) was measured with a BLM. This study discusses the uniformity of the arrangement type, and the size and density of various microstructures the LGP to design suitable microstructures of the LGP for the microstructural design experiment. Finally, a metal mold insert was fabricated with a suitable microstructure design on LGP to manufacture molded LGPs by μIM.

**Figure 3:**
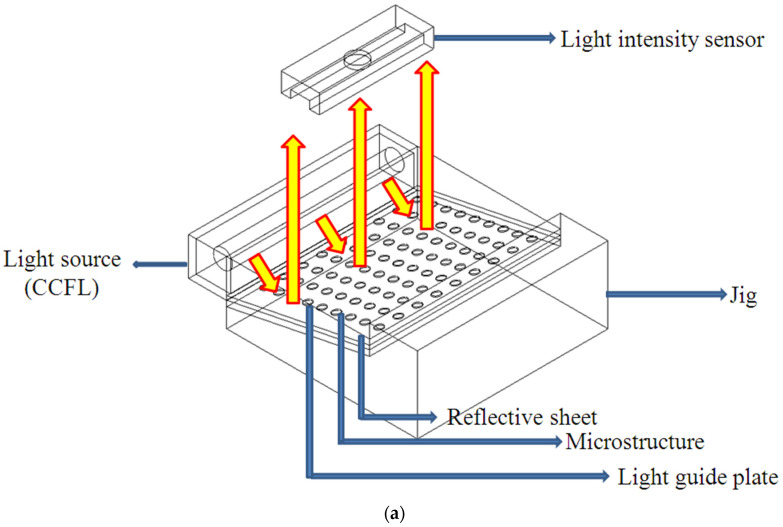

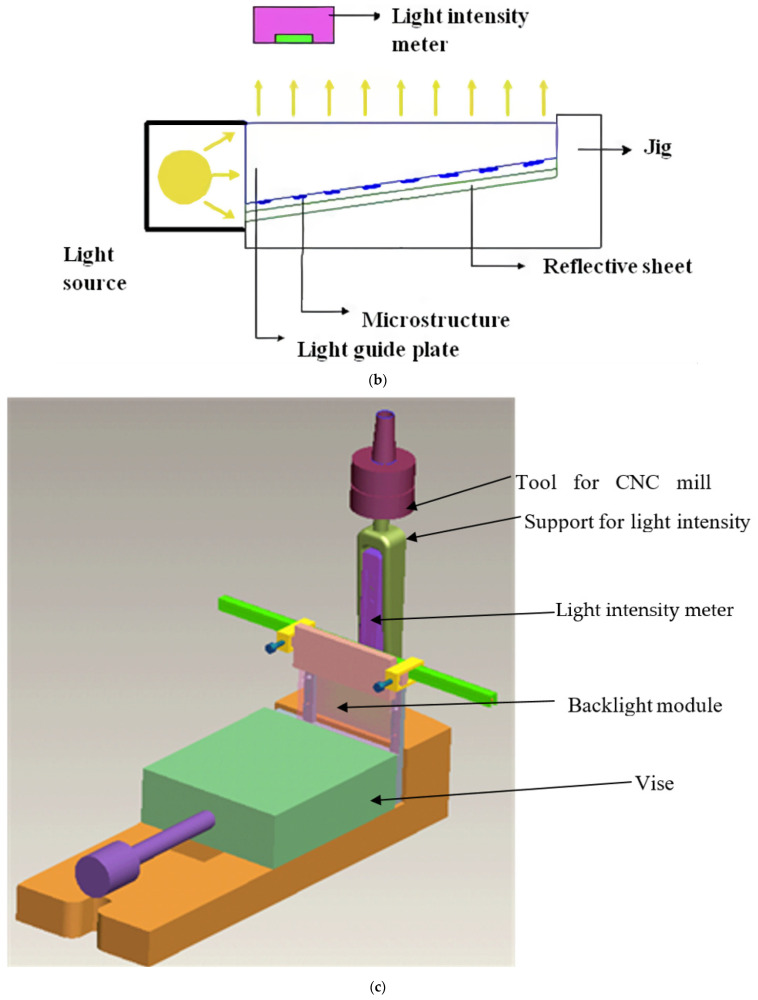
Measurement method of illuminance. (**a**) 3D illustrate; (**b**) 2D Illustrate; (**c**) Real situation for measurement.

**Figure 4:**
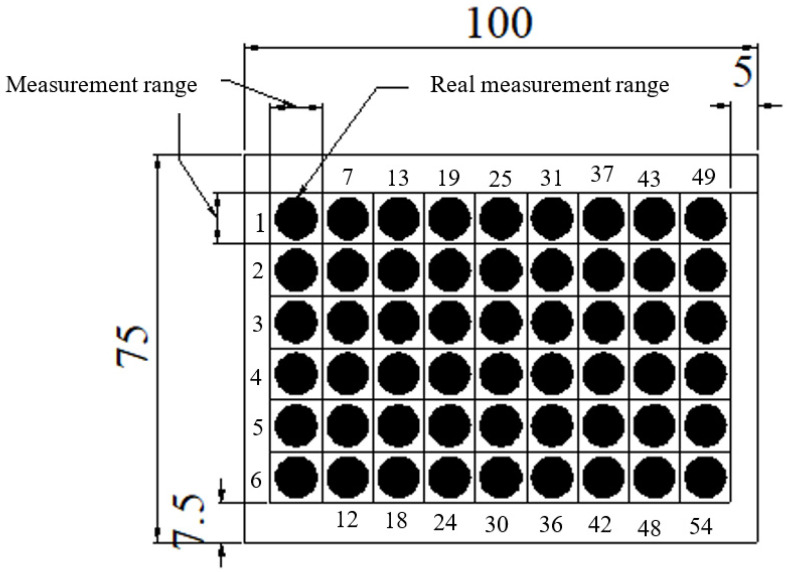
Measurement points of molded light guide plate. (unit = mm).

This study applied a light intensity meter (LS-110; Konica, Tokyo, Japan) to assess the illuminance distribution of the LGP with microstructures. The measurement utilized a point-shaped single-lens reflex type non-contact measurement digital luminance meter; the optical system was an 85 mm f/2.8 lens and a single-lens reflex observation system and the measurement range was FAST at 0.01~999,900 cd/m^2^ and SLOW at 0.01~499,900 cd/m^2^. Texture developed by the authors was used to fix the BLM (i.e., the LGP, reflective sheet, diffusive sheet, prism sheet, and CCFL on the measurement platform (i.e., the x-y table)). A light intensity meter was located on the *z*-axis of the measurement platform to capture the illuminance field of the LGP. Figure 3 displays the measurement system for the illuminance field of the LGP. Because the light intensity meter used in this experiment to measure the uniformity could just count the amount of monochromatic light the authors copied the diffusion points to the blue color for the slide and the light tube of the light source (CCFL) was adhered to the blue paper. Finally, the authors adjusted the light intensity meter to measure the wavelength of blue light (453 nm) to ensure the measurement accuracy. Figure 4 reveals the measurement points for the illuminance distribution of the LGP with microstructures. In this study, we measured the uniformity of the light emitted from the front of the LGP through the BLM set up by the luminance meter in the research institute. This experiment divided the entire surface of the LGP into 9 × 6 equal measurement points on the measurement range. During luminance measurements, since the luminance measurement range was 8 mm, the actual measurement area needed to be corrected, so each measurement value had to be multiplied twice (from the point of view of the measurement area, the measurement range was 10 mm × 10 mm = 100 mm^2^, but the actual measurement range is 4 mm × 4 mm × 3.1415 = 50.2654 mm^2^, so it had to be multiplied twice). Uniformity of the light-emission of the entire surface of the tapered LGP was not achieved, which meant that there was an error of each measurement point to ensure uniformity. The value of each measurement point was calculated using the standard deviation and is expressed as the uniformity, and the signal-to-noise (S/N) ratio was calculated using the small characteristic of the Taguchi method to determine the important parameters that influenced the uniformity.

**Figure 5:**
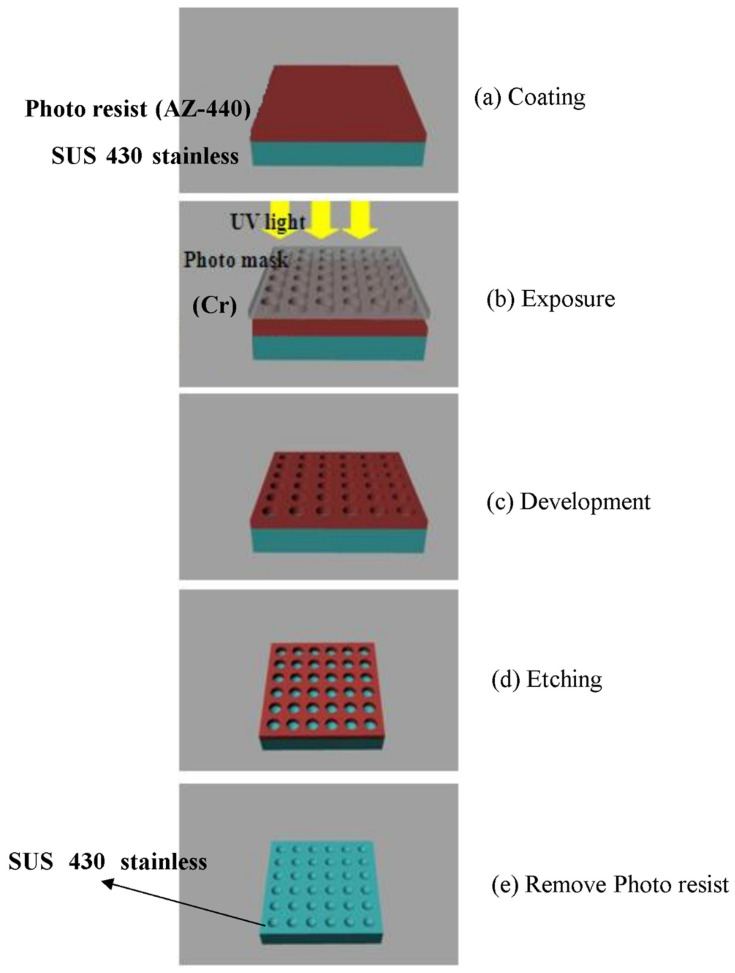
Processing chart for photolithography method of mold insert.

The LGP mold insert with its patterned surface of microstructures was fabricated by a photolithographic method. Fabrication of the mold insert was conducted as follows. The size of the mold insert was 100 mm × 75 mm; the thickness of the thick end was 2.8 mm, and that of the thin end was 0.8 mm. Its microstructure was semi-spherical, 57.6 μm high, and with a diameter of 100–300 μm from the thick end to the thin end by linear expansion. The mold insert, made of SUS 430 stainless steel, was fabricated by a photolithographic method. Figure 5 shows the photolithographic process chart. First, the steel mold insert was cleaned and degreased. Chromium (Cr) was used to design and fabricate the photo mask from the original pattern design. The AZ-440 photo resist was then coated onto the surface of the molded steel with a spin coater. A UV light source was applied to expose the photo-resist through a photo mask. After this exposure process, the photo resist was developed. FeCl_3_ liquid was then employed to etch the mold insert where no photo-resist existed. Finally, residual photo resist was removed and the mold insert was cleaned. An optical microscope (Vertx 220; Micro-VU, Windsor, CA, USA) was used to measure the mold insert with microstructures, and its resolution was 0.5 μm. A commercial IM machine (CLF-125T; Chen Hsong, Zhongli, Taiwan) was applied in all experiments; its screw diameter was 40 mm and clamping force was 125 tons during the μIM process. The mold temperature control machine was a Regloplas 300S (St. Gallen, Switzerland). Its temperature range was 20~140 °C, and its precision was ±1 °C. Optical-grade PMMA (Delpet 80NH; Asahi, Tokyo, Japan) material was employed for μIM. Identifying the effects of various process parameters on the optical quality of the molded LGP was extremely important. Five μIM process parameters of the mold temperature, melt temperature, injection pressure, packing pressure, and packing time were selected as factors for evaluation. Table 3 indicates the process parameters and parameter levels selected for the principal experiment on μIM.

This study characterized the illuminance field distribution of the molded LGP with various μIM process parameters. The molded LGP with microstructures was applied to an LCD in this study. The uniformity of light generated by the molded LGP is very important for LCDs. The illuminance field distribution and uniformity of the molded LGP were the focus of the optical analysis.

## 3. Results and Discussion

**Figure 6:**
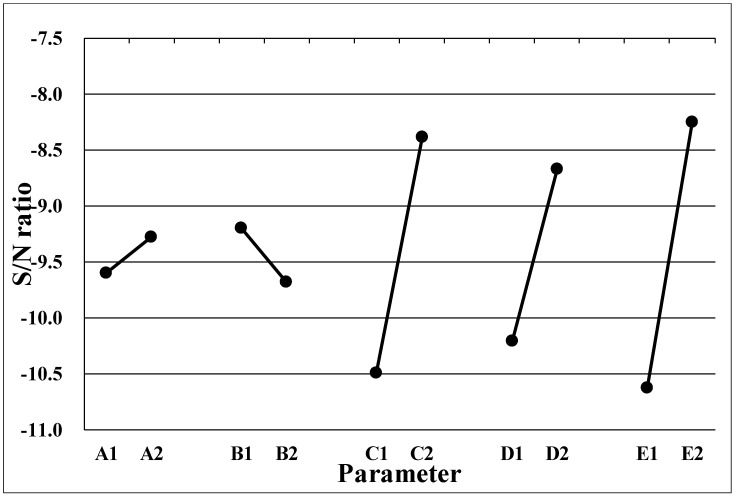
The S/N reaction diagram of uniformity of light guide plate with microstructure.

**Figure 7:**
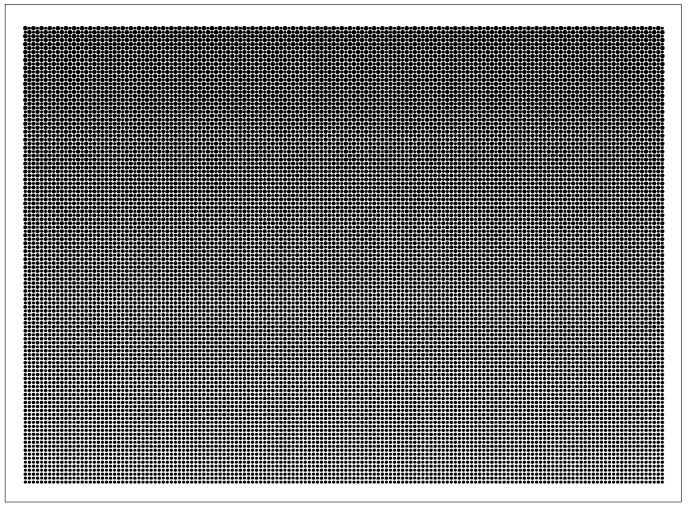
The ultimate microstructure distribution of light guide plate.

The surface area of the LGP with a pattern distribution was 6494.0797 mm^2^, and the area occupied by the pattern distribution was 2960.334 mm^2^. Therefore, the pattern distribution density was equal to 2960.334/6494.0797 = 0.456. To maximize the optimal design for uniformity of the LGP with microstructures, the following equation was employed for the analysis to describe the bigger-the-better characteristics:

(1) $$\frac{S}{N}=-10\log\{\frac{1}{n}\sum_{i=1}^{n}\frac{1}{y_{i}^{2}}\}$$

where *y_i_* is the measured property (uniformity), and *n* corresponds to the number of samples in each test trial. Table 4 demonstrates the *S/N* ratio for the optimal design of the LGP with microstructures. We created an *S/N* reaction diagram of uniformity of the LGP with microstructure (Figure 6). Optimal levels of factors that statistically resulted in the maximum uniformity of the LGP with microstructure were predicted to be A2B1C2D2E2. These results mean that the distance in the ΔX-direction (A) was 1 mm, that of the ΔY-direction (B) was 0.875 mm, existence of insertion point (C) was Φ0.2 mm, the size of the circular shape of the diffusion point at the intersection point of the thick end (D) was Φ0.35 mm, and the size of the circular shape of the diffusion point of thick end (E) was Φ0.35 mm. The most important factor of the design parameters was the size of the circular shape of the diffusion point at the thick end, followed by the existence of the insertion point, and the size of the circular shape of the diffusion point of the intersection point at the thick end; the distance in the ΔY-direction and the distance in the ΔX-direction were unimportant factors. The reason is that a small distance can enhance the distribution density of the microstructure of the LGP. If these distances are too small, they have limited influence on the distribution density. Design parameters of C, D, and E directly influenced the distribution density of the LGP with microstructures. Thus, these parameters significantly influenced the illuminance of the LGP. Figure 7 indicates the ultimate microstructure distribution of the LGP. The goal of the optimal LGP design with microstructures was to achieve good uniformity to the LCD.

**Figure 8:**
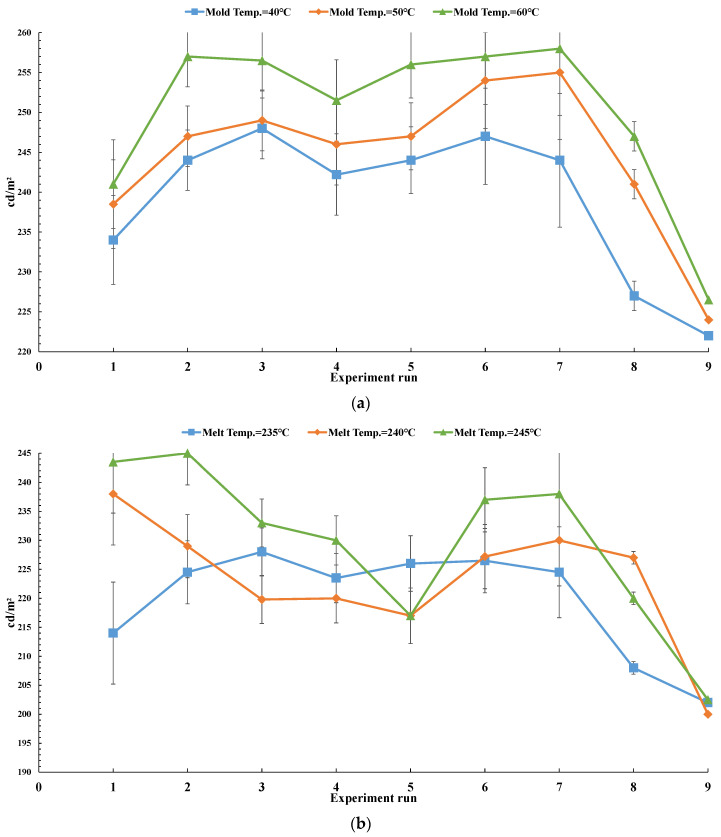

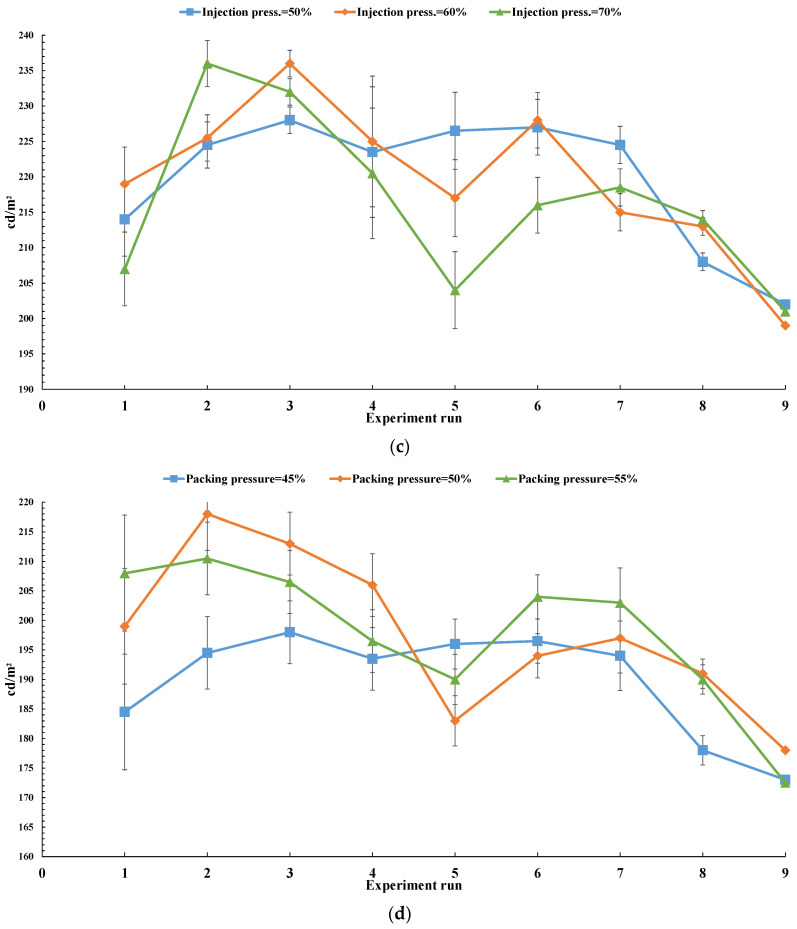

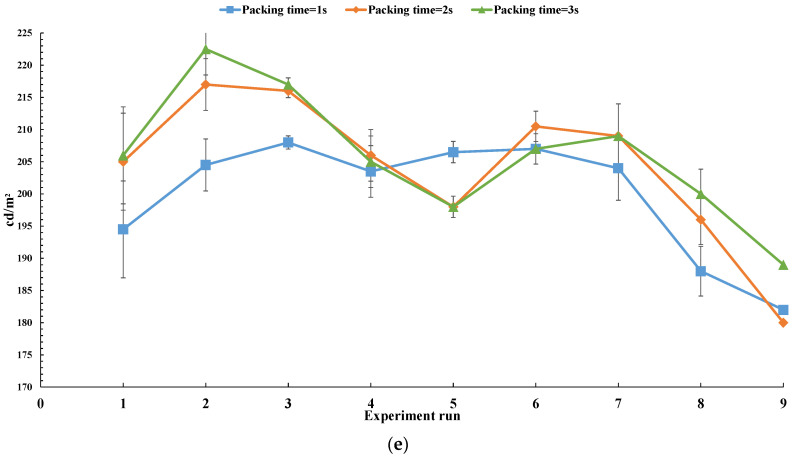
The ultimate microstructure distribution of light guide plate. (**a**) illuminance vs. mold temperature; (**b**) illuminance vs. melt temperature; (**c**) illuminance vs. injection pressure; (**d**) illuminance vs. packing pressure; (**e**) illuminance vs. packing time.

Figure 8 displays the illuminance of the LGP with microstructures with various processing parameters during μIM. The uniformity measurement applied a light intensity meter to assess 54 points on the LGP to measure its uniformity. Figure 8a indicates the luminance values of the molded LGP by μIM at various mold temperatures. The measurement as seen from points 1~6 (Figure 6) were close to the CCFL light source, so the measured illuminance values were higher. The illuminance values gradually decreased as one moved closer to the center of the LGP. Illuminance values at points 7, 13, 19, 25, 31, 37, 43, and 49 and at points 12, 18, 24, 30, 36, 42, 48, and 54 were relatively low. A possible reason is that the light source was reflected to the center when it reached the sides. Some of the light at the sides was lost, and the illuminance at the center also became more uniform, and the illuminance at the sides was relatively low. It can be seen from Figure 8a that the higher the mold temperature, the higher the illuminance value of the LGP. Figure 8b shows illuminance values of the molded LGP by μIM at different melt temperatures. It can be seen that the illuminance value at the light exit was relatively high. In the single-parameter method, results are discussed for different melt temperatures, and it was found that the higher the melt temperature, the better the improvement in the illuminance value. Illuminance values of the molded LGP by μIM at various injection pressures are given in Figure 8c. It shows that the illuminance values were the same as the above situation; illuminance values in the center were more uniform, and those on the sides were relatively low. As to the influence of different injection pressures on the illuminance value, the trend of the illuminance value slightly differed from the microstructure transferability. The reason may be the interaction of flatness and microstructure transferability, which affected the distribution of illuminance values. Figure 8d reveals the illuminance values of molded LGP by μIM with various packing pressures. The higher the packing pressure, the higher the illuminance was, but the average illuminance value was the highest at an 80% packing pressure. This trend was consistent with the microstructure transcriptability. Figure 8e shows illuminance values of the molded LGP by μIM at various packing times. It can be seen that the packing time did not greatly affect the illuminance value. To sum up, the mold temperature was the most important process parameter for the illuminance of the LGP during μIM, followed by the injection pressure and melt temperature. The packing time and packing pressure were unimportant process parameters for the illuminance of the LGP during μIM.

**Figure 9:**
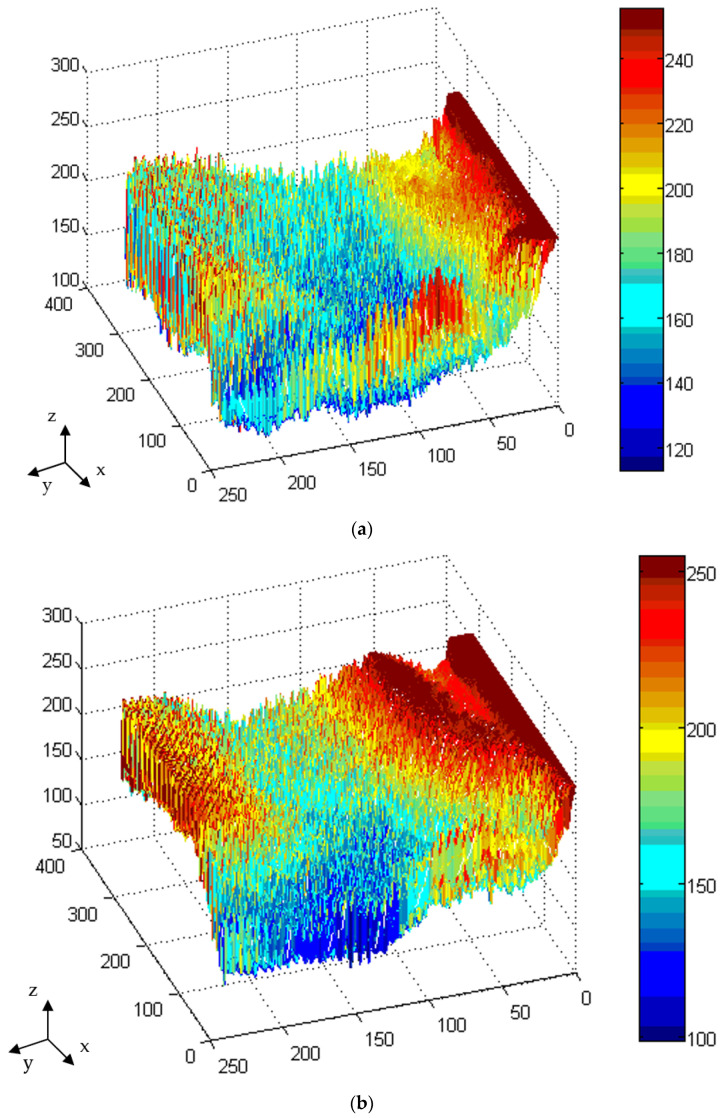

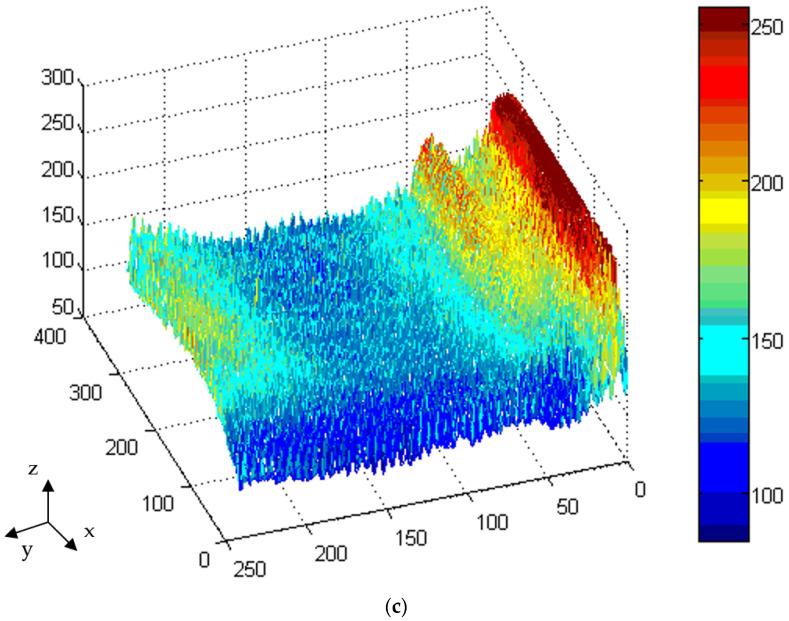
The illuminance of light guide plate with and without microstructure. (**a**) Light guide plate without microstructure; (**b**) Light guide plate with microstructure for primary design; (**c**) Light guide plate with microstructure for optimal design.

Figure 9 exhibits the illuminance field distribution of the LGP with and without microstructure fabricated by μIM. The light intensity at the light entrance was the brightest. The light intensity decreased as one moved away from the light source, while the light intensity increased at the end where the light traveled. The entire measurement trend was available (in terms of the *Y*-axis), and the trend was: strongest → strong → uniform → uniform → uniform → strong. The reason for this distribution is that the light input was the light source, so the measured value was largest. When the light traveled to the middle part, the measured value was more uniform due to the effect of the microstructure design of the LGP, because the light was sealed in the installed BLM. When the light traveled to the module end, it was reflected back, so at the module end, the original light intensity should be weak due to reflection (because the light was lost during travel), but the light intensity was strong. The illuminance field distribution of the LGP without microstructures (Figure 9a) had minimum illuminance (compared to Figure 9b,c). The reason is that this LGP could not reflect light and thereby could not enhance the illuminance. The average illuminance of the LGP without microstructures was roughly 160.2 cd/m^2^. Figure 9b reveals the illuminance field distribution of the LGP with microstructures and the primary design. Figure 9b shows that the illuminance field distributions of the LGP with microstructures were larger than the illuminance of the LGP without microstructures (Figure 9a). The average illuminance of the LGP with microstructures and the primary design was roughly 175.4 cd/m^2^. The illuminance field distribution of the LGP with the optimally designed microstructures is demonstrated in Figure 9c. The illuminance field distribution of the LGP with optimally designed microstructures had the maximum illuminance (compare Figure 9a,b). The average illuminance of the LGP with optimally designed microstructures was roughly 196.1 cd/m^2^. Its average illuminance was 1.3 times that of the LGP without microstructures. The illuminance obtained in this study was better than the results of a reference designation [19]. The illuminance of the LGP revealed the maximum value near the inlet of the light source. The illuminance then decreased as the position moved away from the light source. Finally, the illuminance increased at the last position the light moved. The illuminance of the LGP changed from strong to weak and to strong again as the distance increased between the LGP and the light source (in the Y-direction). The illuminance of the LGP reached a maximum value at a position close to the light source, and this light did not decay. The illuminance of the LGP decreased in the middle region of the LGP because the light had decayed as it traveled away from the light source. Finally, the light arrived the final position of the LGP and was reflected back into the LGP, such that the illuminance of the LGP increased.

The uniformity of the output light is the ratio of the minimum illuminance (I_min_) to the maximum illuminance (I_max_) at the measured points on the observation plane, and is expressed as

Uniformity (U) = (I_max_ − I_min_)/I_max_ × 100% (2)

Figure 9 shows the uniformity of the LGP with and without microstructures fabricated by μIM. The LGP without microstructures (Figure 9a) had minimum uniformity (compared to Figure 9b,c) because this LGP could not reflect light and thereby enhance the uniformity. The average uniformity of the LGP without microstructures was roughly 52.4%. Figure 9b shows the uniformity of the LGP with microstructures on the primary design. Figure 9b indicates that the uniformity of the LGP with microstructures was larger than that of the LGP without microstructures (Figure 9a). The uniformity of the LGP with microstructures on the primary design was roughly 66.8%. The uniformity of the LGP with microstructures on the optimal design is demonstrated on Figure 8c. The uniformity of the LGP with microstructures and the optimal design had the maximum uniformity (compare Figure 9a,b). The uniformity of the LGP with microstructures and the optimal design was roughly 86.4%. Its uniformity was nearly 1.65 times that of the LGP without microstructures. The uniformity obtained by this result was better than the result in several references [13,23,27,31,46] and was slightly less than results of other references [14,21,24,37,45]. The authors made comparisons with previous results, and this study optimized the design of the microstructures of the LGP, the uniformity of which could be the most effective BLM application.

**Figure 10:**
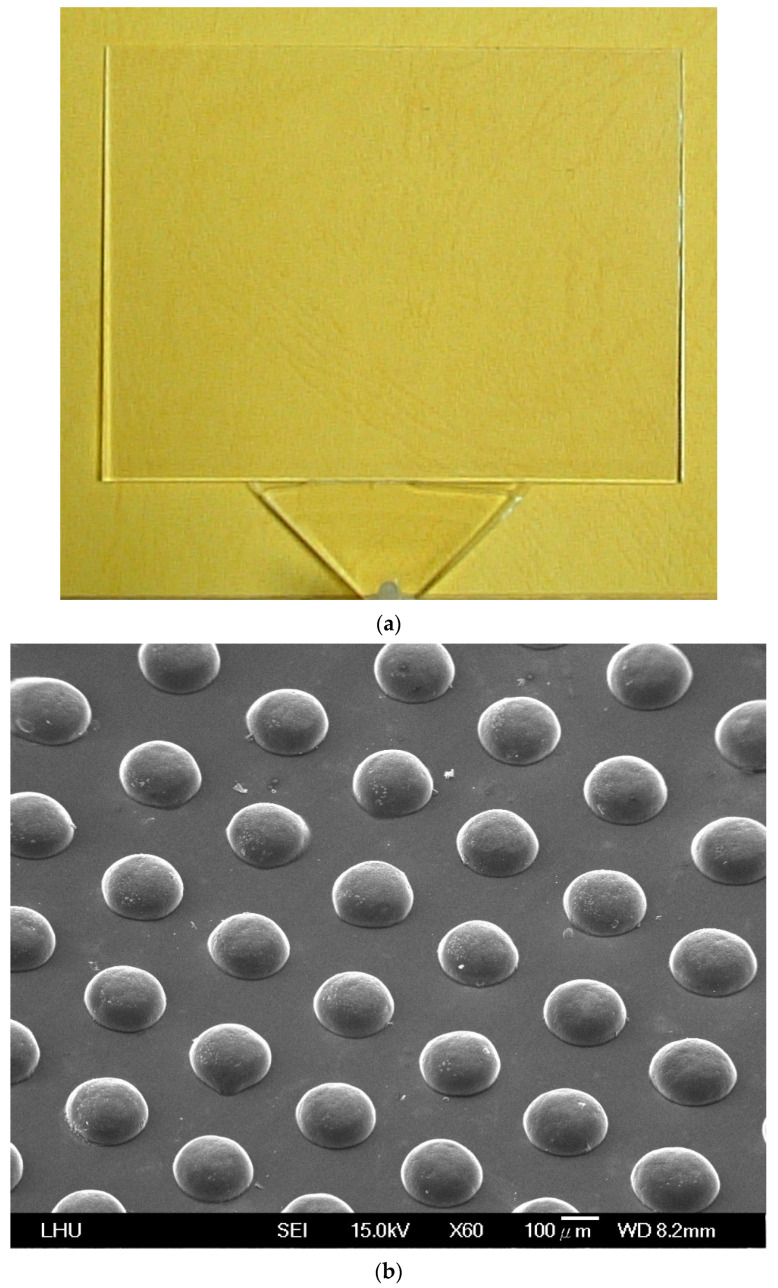

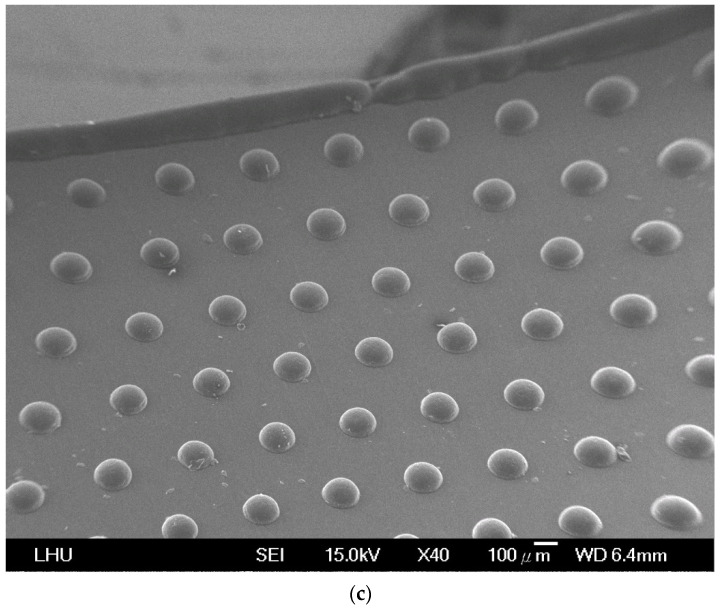
The molded light guide plate. (**a**) Light guide plate; (**b**) Microstructure on thin end; (**c**) Microstructure on thick end.

Figure 10 shows the molded LGP and its microstructures used in this study. Figure 10a shows the molded LGP. The microstructures of the LGP were fabricated very well from the thick end to the thin end (Figure 10b,c, SEM image). The results evidence that this study achieved a good LGP from the microstructure design, mold design and fabrication process to make an optimal the LGP by μIM.

## 4. Conclusions

This study applied a design method for fabricating a mold insert for an LGP with microstructures using a photolithographic method. Then, μIM processing was used to successfully fabricate a molded LGP with microstructures transferred from the mold insert. We successfully designed and manufactured an actual molded LGP with microstructures. In order to achieve the optimal uniformity of the illuminance field distribution for processing parameters on μIM, we applied various processing parameters-mold temperature, melt temperature, injection pressure, packing pressure, and packing time-to fabricate the molded LGP by μIM. When we manufactured the molded LGP by μIM, as the mold temperature changed, the illuminance value of the LGP with microstructures reached the highest value (257 cd/m^2^). The mold temperature was the most important factor influencing the illuminance field distribution of the molded LGP with μIM. The packing pressure and packing time were unimportant process parameters for the illuminance field distribution of the molded LGP during μIM. The molded LGP with microstructures under the optimal design had better uniformity (86.4%) than that with microstructures under the primary design (66.8%) and that without microstructures (52.4%). Under the optimized design, the uniformity of the molded LGP with microstructures was 1.65 times that of the molded LGP without microstructures. Therefore, an optimized LGP with microstructures had good results for the uniformity of the illuminance field distributions in this study.

## Figures and Tables

**Table 1:** Values of stagger and insertion pattern of diffusion points of microstructure of light guide plate.

	Level	I	II
Factor			
A. Distance of △X direction		0.875	1
B. Distance of △Y direction		0.875	1
C. Existence of insertion point		none	0.2
D. Size of circular shape of diffusion point at the intersection point of thick end		0.2	0.35
E. Size of circular shape of diffusion point of thick end		0.2	0.35

**Table 2:** Experiment for stagger and insertion type array.

	Factor	A	B	C	D	E
Runs						
1		0.875	0.875	none	0.2	0.2
2		0.875	0.875	none	0.35	0.35
3		0.875	1	0.2	0.2	0.2
4		1	0.875	0.2	0.35	0.35
5		1	0.875	0.2	0.2	0.35
6		1	0.875	0.2	0.35	0.2
7		1	1	none	0.2	0.35
8		1	1	none	0.35	0.2
9		Without microstructure				

**Table 3:** Parameters and levels selected in the main experiment of μIM.

Parameters	Level 1	Level 2	Level 3
a. Mold temp. (°C)	40	50	60
b. Melt temp. (°C)	235	240	245
c. Injection pressure (%)	50	60	70
d. Packing pressure (%)	45	50	55
e. Packing time (sec.)	1	2	3

**Table 4:** S/N ratio for optimum design of microstructure of LGP.

Runs	Average	RMS Deviation	S/N Ratio	Distribution Density
L1	2.791481	3.020900	−12.2830	0.2672
L2	2.333330	1.306883	−8.5445	0.3743
L3	2.485185	2.437346	−10.8330	0.3048
L4	2.025926	0.791949	−6.7500	0.3997
L5	2.248148	0.957230	−7.7600	0.3515
L6	2.262593	1.226099	−8.2100	0.3509
L7	2.615185	1.760603	−9.9730	0.2462
L8	2.732593	2.381861	−11.1860	0.2436
L9	3.085556	6.668369	−17.3230	None
